# Antigen-Presenting Cells and Antigen Presentation in Tertiary Lymphoid Organs

**DOI:** 10.3389/fimmu.2016.00481

**Published:** 2016-11-07

**Authors:** Catherine E. Hughes, Robert A. Benson, Marija Bedaj, Pasquale Maffia

**Affiliations:** ^1^Centre for Immunobiology, Institute of Infection, Immunity and Inflammation, College of Medical, Veterinary and Life Sciences, University of Glasgow, Glasgow, UK; ^2^Rheumatology Research Group, Centre for Translational Inflammation Research, School of Immunity and Infection, College of Medical and Dental Sciences, University of Birmingham, Birmingham, UK; ^3^BHF Centre of Excellence in Vascular Science and Medicine, College of Medical, Veterinary and Life Sciences, University of Glasgow, Glasgow, UK; ^4^Department of Pharmacy, University of Naples Federico II, Naples, Italy

**Keywords:** antigen presentation, antigen-presenting cells, B cells, dendritic cells, follicular dendritic cells, macrophages, tertiary lymphoid organs

## Abstract

Tertiary lymphoid organs (TLOs) form in territorialized niches of peripheral tissues characterized by the presence of antigens; however, little is known about mechanism(s) of antigen handling by ectopic lymphoid structures. In this mini review, we will discuss the role of antigen-presenting cells and mechanisms of antigen presentation in TLOs, summarizing what is currently known about this facet of the formation and function of these tissues as well as identifying questions yet to be addressed.

## Introduction

The ability to respond rapidly and effectively to damage or infection is mediated by the immune system. Secondary lymphoid organs (SLOs) such as lymph nodes (LNs) and spleen provide critical meeting points for immune cells and antigens, promoting interactions that result in a prompt, targeted immune response. A key process in initiating and sustaining such an adaptive response is in the delivery of antigen for interrogation by lymphocyte populations. Networks of lymphatic vessels channel free and cell-borne antigen to the lymph node where it is then further directed to appropriate lymphocyte compartments through additional structural and cellular filters. However, during chronic inflammation, ectopic lymphoid tissue can form in the periphery, outside the normal sites of secondary lymphoid organogenesis. This tissue shares common features with SLOs, including segregated T and B cell areas, germinal centers (GCs), development of structural stromal components, and vascularization, with high endothelial venules (HEVs) often observed (1). These so-called tertiary lymphoid organs (TLOs) are thought to function as a local site for perpetuation of adaptive immune responses providing a local source of antibody, generated as the result of local antigen presentation, and lymphocyte activation and maturation in the newly formed structure. In some settings, development of these lymphoid structures may be advantageous, as in bacterial and viral infections (2–4), atherosclerosis (5, 6), and cancer (7, 8), while in a number of diseases, particularly autoimmune disorders, the development of TLOs may be associated with non-resolving inflammation, with a vigorous and sustained response to self-antigen amplifying severe and chronic pathology [reviewed in Ref. (9); see also Ref. (10–12)]. In this review, we will outline the role of antigen-presenting cells (APCs) and mechanisms of antigen presentation in the TLO, summarizing what is currently known about this facet of the formation and function of these tissues as well as identifying questions yet to be addressed.

A spectrum of TLO development has been documented in the literature, with ectopic lymphoid tissue ranging from relatively loose aggregates of B and T cells to highly compartmentalized, complex structures that include stromal scaffolding supporting distinct T cell zones and secondary B cell follicles containing GCs, i.e., *bona fide* lymphoid organs with clear parallels to secondary lymphoid tissue (9, 13). The initial steps leading to development of TLOs are unclear, as a number of distinct but inter-related signals can prompt development of organized lymphoid structures in non-lymphoid tissue. Determining the “tipping point” beyond which development of functional, relatively stable ectopic lymphoid tissue is inevitable is inherently difficult. However, a number of features are known to affect formation and stability of the structure. Several studies, described in more detail elsewhere in this research topic issue, variously indicate increased expression of cytokines such as lymphotoxin (LT) and other TNF family members (14), IL-22 (15), and chemokines such as CXCL13 and CCL21 (16–18) as being capable of inducing TLO formation. In a patho-physiological setting, specific cell types (19), including T cells (16), and APCs, such as macrophages (20), dendritic cells (DCs) (2, 3), and activated B cells (21), are all described as possible key players in early expression of cytokines and chemokines that promote increased tissue infiltration by leukocytes, development of lymphoid stromal cells such as follicular dendritic cells (FDCs), and construction and maintenance of the functional TLO. Fluid accumulation at the site of infection has also been suggested to influence TLO development (22).

## Antigen-Presenting Cell Populations within TLOs

### Dendritic Cells

Although an ever-increasing number of cell types have been shown to be capable of presenting antigen to immune cells, the classical professional antigen-presenting cell is the conventional dendritic cell (23).

Their involvement in various types of TLO has been demonstrated by a number of studies. In a model of viral lung infection, Halle et al. (2) showed that early infiltration of CD11c^+^ cells into the perivascular and peribronchiolar space (4 days post infection) precipitated recruitment of lymphocytes to the infected tissue, with subsequent development of organized inducible bronchus-associated lymphoid tissue (iBALT) structures. Within these highly developed structures, DCs resided primarily within the T cell area, as in SLOs. When CD11c^+^ cells were selectively depleted at various time points using a diphtheria toxin receptor (DTR) transgenic model, the size, but not frequency, of iBALT was reduced, suggesting an important role for DCs, and possibly alveolar macrophages, in maintaining TLO integrity (2). A concurrent study, investigating induction of iBALT in a model of influenza infection, also demonstrated a key role for CD11c^+^ cells in maintenance of these lymphoid structures. Again, using a DTR-transgenic model, this study showed that selective depletion of CD11c^+^ cells from lungs with mature iBALT led to disintegration of the TLO and gradual dispersal of lymphocytes from the lung (3). Notably, influenza-specific plasma cells were found to be undetectable soon after DT-induced depletion of CD11c^+^ cells, while total B cells and peanut agglutinin (PNA)^+^ GC B cells were also substantially reduced. The level of class-switched immunoglobulin, specifically IgA, was also significantly reduced in bronchoalveolar lavage fluid. These results indicate a prominent role for DCs in the function and maintenance of iBALT following influenza infection, as well as suggesting an important role for the TLO in local production of class-switched antibodies. Somewhat surprisingly, depletion of CD11c^+^ cells also led to a significant reduction in the level of systemic hemagglutinin-specific antibody present, indicating a potential role for TLO GCs in generation of long-lived plasma cells that home to the bone marrow (BM). To investigate the role of antigen presentation by DCs in this tissue, lung DCs were isolated from animals challenged with influenza virus expressing the MHC-II OVA_323–339_ epitope, at days 4 and 17 post infection. While these DCs were able to activate OVA-specific CD4^+^ T cells (OT-II) at day 4, this was no longer the case at the later time point. However, they retained antigen-presenting ability, as demonstrated by DC-mediated activation of OT-II cells after addition of pre-processed OVA peptide. The authors suggest that the primary role of the DC population in maintenance of the iBALT is production of LTβ, which in turn induces high levels of CXCL13, an important chemokine in B cell migration and retention. Finally, the study also demonstrated that adoptive transfer of granulocyte-macrophage colony-stimulating factor (GM-CSF)-cultured BM-derived cells (a mix of conventional DCs and monocyte-derived macrophages) intratracheally into the lungs of naïve mice leads to iBALT development (3).

In a model of thyroid TLO development, where high levels of CCL21 were artificially induced in the thyroid, CD3^+^CD4^+^ T cells from an adoptively transferred mixed splenocyte population were found to be the initiating cell type in development of ectopic lymphoid tissue. Subsequent recruitment of host DCs and DC/T cell interactions were found to be important for the formation of peripheral-node addressin-positive (PNAd^+^) HEVs in the developing TLO, in a LTα-/LTβR-dependent manner (16). A subsequent paper by the same group confirmed that depletion of CD11c^+^ DCs led to reduced lymphangiogenesis in the thyroid (24).

LTβR is known to have an important role in the maintenance of HEVs within LNs (25), again indicating that these structures are *bona fide* lymphoid organs. Interestingly, in a model of insulin-dependent diabetes mellitus induced by adoptive transfer of specific antigen-expressing DCs, only mice that showed early infiltration of leukocytes and formation of islet-associated organized lymphoid structures in the pancreatic parenchyma went on to develop diabetes, suggesting a link between antigen presentation by DCs to T cells, TLO formation, and development of autoimmunity (26). More recently, the presence of mature DCs in tumor TLOs was highly associated with a favorable clinical outcome in patients with lung cancers (27, 28); however, to date, there is no direct demonstration that APCs in TLOs permit efficient local T-cell priming against tumor-associated antigens.

### Macrophages

Macrophages are some of the earliest immune cells to encounter antigen at sites of infection or injury. The response to antigenic stimulus is context-dependent but production of inflammatory cytokines is a key function of these cells in the early stages of inflammation. In the case of atherosclerosis, macrophages that infiltrate the early plaque take up oxidized low-density lipoprotein (ox-LDL) particles and are activated, including upregulation of antigen-presentation genes and increased production of inflammatory cytokines (29). Recently, work from Guedj et al. has proposed a role for pro-inflammatory macrophages as a kind of lymphoid tissue inducer (LTi) cell in the development of artery tertiary lymphoid organs (ATLOs) during atherosclerosis. In this study, BM-derived macrophages were incubated with both LPS and IFNγ to yield a “pro-inflammatory” phenotype or with IL-4 to generate “alternatively activated” macrophages. Vascular smooth muscle cells (VSMCs) incubated with LPS/IFNγ-stimulated macrophages, which produced TNFα and LTα, developed a lymphoid tissue organizer (LTo) phenotype, while those incubated with IL-4-stimulated macrophages did not (20). This activity did not require LTβR signaling but was dependent on TNF receptor involvement. In addition, Jupelli et al. (4) have reported that iNOS-expressing macrophage involvement in early stages of bacterial lung infection precedes development of iBALT in the lungs of infected mice in their model. Intratracheal transfer of “pro-inflammatory” macrophages (generated from BM-derived macrophages cultured with IFNγ) into infected lungs leads to increased lung inflammation and iBALT formation. It should be also noted that, although the recruitment of CD11c^+^ DCs is clearly a crucial step in the development of iBALT in the viral infection model from Halle et al. (2), the earliest infiltrate recorded was that of alveolar macrophages, within 5–7 h of infection, with DC accumulation described from 4 days post infection. As TLOs, by their nature, form during inflammatory events, and particularly during sustained inflammation, it is logical to assume that macrophage production of inflammatory cytokines following antigen encounter is a necessary, but probably not sufficient, primary event in TLO formation. As described for ATLO formation, a possible role for macrophages as a type of inducible LTi remains to be demonstrated for other types of ectopic lymphoid tissues.

### B Cells

The main role of B cells in an immune response is production of antibodies. B cell presentation of antigen to T cells is an integral aspect of this function. These interactions allow B cells to receive survival signals and direct them appropriately to generate high affinity antibody specific to the antigen encountered (30). Well-established, highly organized TLOs contain secondary B cell follicles, which form following antigen encounter and activation of B cells (31–33). The GCs of these follicles are structurally and functionally similar to those within SLOs, with FDC development described within a number of ectopic lymphoid tissues. This lends credence to the hypothesis that TLOs provide a venue for local production of antibody proximal to the site of inflammation, with either beneficial (e.g., during infection, cancer, or atherosclerosis) or deleterious (e.g., autoimmunity) effects depending on the context in which the TLO forms.

B cells are involved in FDC development (34) in a LT- and TNF-dependent manner, with B cell aggregates shown to induce FDC through LTα1β2 production in SLOs (35, 36). LTα1β2 expression by naïve B cells is induced by CXCL13 (also known as B-lymphocyte chemoattractant), which is itself induced by LTα1β2 in a positive feedback loop (37), with FDCs likely the major source of CXCL13 in the follicle (36). In mice with B cells lacking LTβ, FDC structures in the spleen were disrupted, though not wholly absent (13). Similarly, LTα is not fully required for formation of iBALT, as lymphocytic aggregates form in influenza-infected *Lta^−/−^* mice and lymphoid chemokines CXCL13 and CCL21 are detectable. However, these structures lack the level of development and organization of the TLO observed in mice expressing LTα (17). A similar role in promoting FDC formation has been suggested for B cells in TLOs that arise in the salivary glands of Sjögrens syndrome patients (38).

### Follicular Dendritic Cells

Follicular dendritic cells are cells of the immune system found in B cell follicles. FDCs are integral to the function of the follicle, presenting antigen in the form of immune complexes bound to their surface (39, 40). FDCs are believed to provide a uniquely long-lasting “depot” of antigen that can be accessed by B cells well beyond clearance of the initial infection or injury from which the antigen was acquired. They are thought to be important in the affinity maturation of the B-cell receptor (BCR). Only B cells expressing a receptor of high enough affinity will be successful in acquiring sufficient antigen from the FDC to in turn present the antigen to their cognate T cell and receive survival signals (36). A recent study has shown that disruption of the FDC network in a model of arthritis led to reduced GC formation in lymphoid follicles, impaired recruitment of follicular helper T (Tfh) cells into B cell areas, diminished autoantibody production, and attenuation of disease (41).

Although there is some debate over how B cells within a TLO might perceive antigen due to the likelihood of increased availability of local antigen compared to SLOs, a number of studies have identified FDCs within ectopic lymphoid structures (5, 42–45). The source of these cells within TLOs is unclear, but, as discussed above, various studies indicate that B cell production of LTα1β2 is important for differentiation of FDCs within ectopic lymphoid organs (1, 35, 38), even though follicle formation in BALT has been reported also in the absence of differentiated FDCs (46). In addition to providing a platform for antigen presentation, FDCs are also known to produce a variety of cytokines and chemokines involved in B cell migration survival and proliferation, as well as recruitment of Tfh cells into B cell areas, such as CXCL13, BAFF, IL-15, and IL-6 (45, 47). Therefore, a similarly multi-faceted role for these cells in mature TLOs is anticipated.

### Other Antigen-Presenting Cells

As reviewed by Kambayashi and Laufer recently, a number of cells not traditionally considered “professional” APC may nonetheless under specific circumstances be induced to express MHC-II on their surface and have been shown to interact with T cells in an antigen-specific manner (23, 48). In Sjögrens syndrome, salivary gland epithelial cells (SGECs) may play an important role in the presentation of self-antigen. Numerous lines of evidence point to this ability, including expression of co-stimulatory molecules, such as CD80, CD86, and CD40 (49, 50), the ability to express adhesion molecules and human leukocyte antigen (HLA)-DR (51), and the ability to activate antigen-specific T cells (48). Ishimaru et al. also suggest expression of IFNγ by SGECs may be involved in increased expression of MHC-II by these cells (48). Self-antigen presentation by thyroid epithelial cells – indicated by MHC-II expression and an ability to induce T cell activation – was described more than 30 years ago, with the authors suggesting that the cells might preferentially present self-antigen (52). Other non-hematopoietic cells have also been implicated in presentation of self-antigen. In 2010, Cohen and colleagues described a role for lymphatic endothelial cells (LECs) in the induction of peripheral tolerance through autoimmune regulator (AIRE)-independent presentation of self-antigen (53). Additionally, extrathymic AIRE-expressing cells (eTACs) have been identified in pancreatic TLO of non-obese diabetic (NOD) mice (54). The ability of eTACs to induce peripheral tolerance in TLOs is yet to be demonstrated, but expression of AIRE in these cells has been linked to non-canonical NF-κB activation, which contributes to peripheral tolerance induction (55). Finally, fibroblastic reticular cells (FRCs) express and present peripheral tissue-restricted antigen to T cells as part of the peripheral tolerance mechanism, and their ability to stimulate T cells is altered depending on the inflammatory state of the tissue (56). These cell types have also been detected in TLOs, again pointing to roles in directing the immune response that unfolds within (5, 45, 57).

While the presence in TLOs of each of the APCs described thus far has been robustly reported in the literature and across a variety of TLOs, in the vast majority of cases there has been limited or no direct investigation of actual antigen presentation in these tissues. What mechanisms exist to allow TLO-associated APCs access to antigen? Who are the main APCs presenting antigen, what are the nature of the antigens, and what are the ultimate immunological consequences of antigen presentation in TLOs?

## Acquiring Antigen for Presentation

In the case of LNs, DCs carrying antigen acquired directly in a peripheral tissue migrate *via* the lymphatic vessels and enter the subcapsular sinus (SCS). Here, the DCs must traverse from the SCS ceiling to the floor, cross the dense parenchyma, and enter the paracortex. Small antigens can also drain freely through the lymphatic bed to the SCS. These antigens can be accessed by DCs already residing within the LN as they pass through conduits linking the SCS and HEVs (58, 59). A comparable conduit system is present in the follicular regions, allowing similar access to antigen by B cells (60, 61). The follicles themselves are positioned directly adjacent to the SCS and may facilitate B cell acquisition of soluble antigen, potentially draining through SCS pores (62, 63) or presented by SCS macrophages (64–66). Another possibility is that B cells could acquire unprocessed antigen from DCs (67). In this instance, uptake by the DC would likely involve the FcγRIIB receptor, allowing the antigen to remain unprocessed and recycled to the cell surface (68). Larger antigens acquired by non-cognate B cells can be further transported to FDCs in a complement-dependent way.

**Figure 1:**
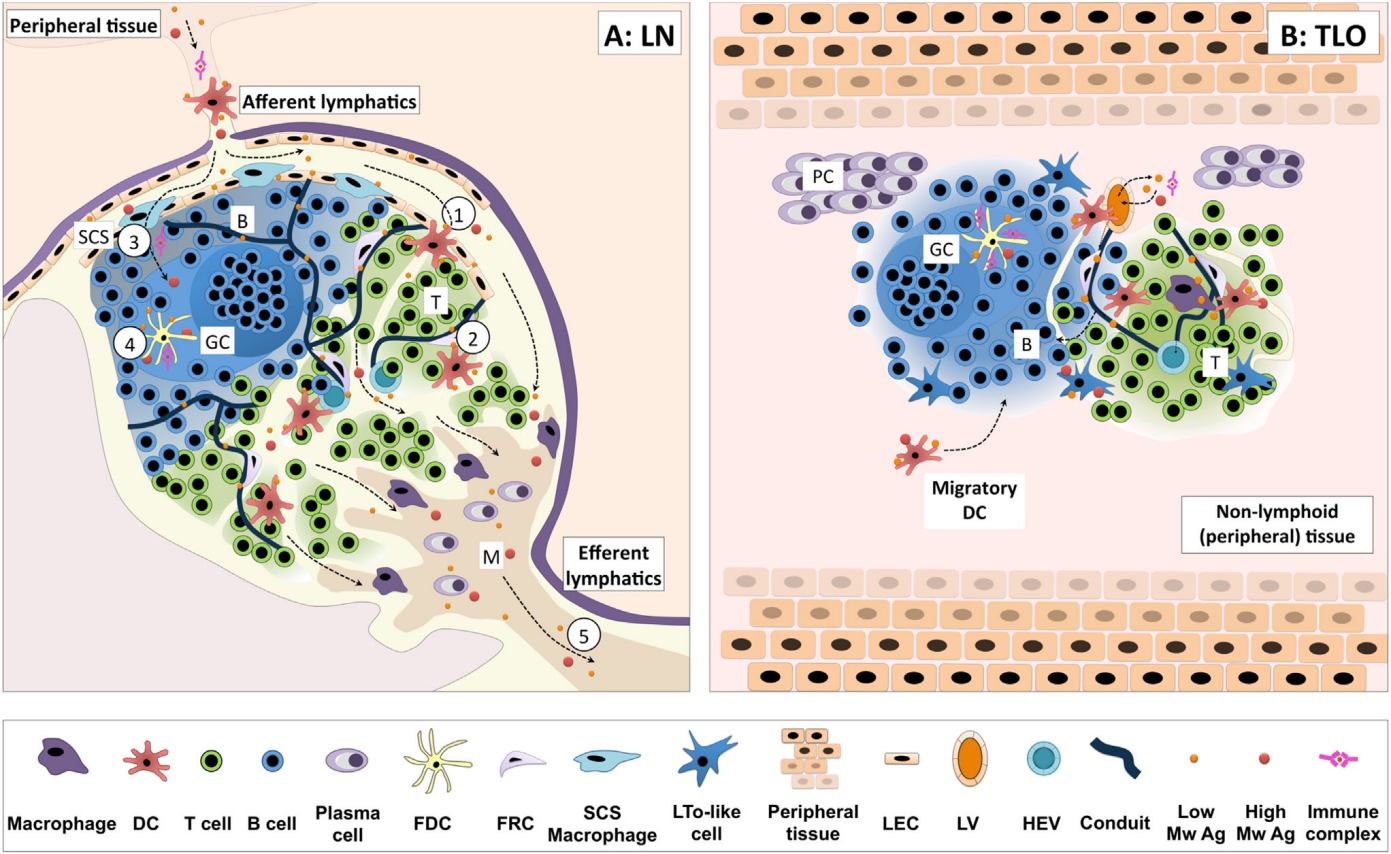
**Antigen handling and presentation in lymph nodes vs. TLOs**. **(A)** Antigen reaches lymph nodes (LNs) *via* the afferent lymphatics, transported by dendritic cells (DCs) or freely draining from the peripheral tissues. Migratory DCs cross the subcapsular sinus (SCS) and enter the paracortex (T cell area), migrating in response to appropriate chemotactic cues (1). Here, they encounter T cells searching for cognate peptide–MHC complexes. DCs residing within T cell areas can also sample small soluble molecules from a network of conduits traversing the paracortex (2). Additionally, small soluble antigens may be accessed by B cells *via* pores in the endothelial layer or transported by SCS macrophages, as is the case for larger antigens and immune complexes (3). Such antigen can be further transferred to FDCs for presentation to B cells (4). Freely draining antigen may also exit the LN efferent lymphatic and reach the next lymph node in the chain (5). **(B)** In general, tertiary lymphoid organs (TLOs) lack a defined capsule, which may allow more free diffusion of antigen through the structure. Lymphatic and conduit-like structures have been identified in TLOs, and may function in an analogous fashion to those in SLOs, although this has yet to be formally demonstrated. Similar cellular compartmentalization is observed between TLOs and SLOs, and they share many common antigen-presenting cell populations. Indeed, the migration of DCs from surrounding tissues to the TLOs has been observed in several models of TLO formation. Thus, TLOs likely share common pathways for handling cell-associated and free antigen with SLOs to optimize the functioning of adaptive immune responses. *Key*: B, B cell follicle; T, T cell area; SCS, subcapsular sinus; GC, germinal center; M, medulla; PC, plasma cell niche; DC, dendritic cell; FDC, follicular DC; FRC, fibroblastic reticular cells; LTo, lymphoid tissue organizer; LEC, lymphatic endothelial cell; LV, lymphatic vessel; HEV, high endothelial venule; low/high Mw Ag, low/high molecular weight antigen.

But what happens in a TLO? To date, direct data pertaining to antigen handling within TLOs is scarce. One might speculate as to the relevance of lymphatics and conduits to antigen transport to/within TLOs since, in general, the majority of TLOs do not demonstrate a distinct capsule or SCS, and form locally at the peripheral site of antigenic challenge. Yet lymphatic vessels do appear to be present in TLOs, such as those seen in ATLOs, iBALT, and pancreatic and thyroid infiltrate (5, 18, 24, 69). But their direct contribution in antigen transport is open for debate [as reviewed in Ref. (22)]. Splenic white pulp lacks afferent lymphatics but demonstrates series of organized cellular transport mechanisms [involving marginal zone macrophages, B cells and DCs (70)] along with a conduit network directly linked to the blood stream (71). As in the LN conduit system, transport of antigen/molecules is similarly size restricted, but does represent one way in which small molecules can be transported to particular compartments within the TLO. ATLOs demonstrate ER-TR7^+^ reticular networks consistent with the presence of conduits. Indeed, conduit structures were seen to extend through the T cell areas terminating adjacent to HEVs of the ATLO. In addition, these conduits were able to channel only small particles (10 kDa) and not larger particles from the adventitia following i.v. administration (5). Evidence of conduit-like structures have been reported in both human and murine studies and in a variety of tertiary lymphoid tissues found in pancreas, kidney, salivary glands, and liver (57). So, it seems likely that an additional contribution of conduits and lymphatics in the instance of TLOs may relate to antigen transport, allowing small molecule percolation throughout the ectopic lymphoid organ, while, as in other lymphoid organs, their major role likely relates to cellular trafficking (transport of chemokines to HEVs in the case of conduits, and perhaps efferent lymphatic functions for removal of inflammatory cells and mediators from the affected tissue). Mechanisms of antigen handling and presentation in LNs vs. TLOs are illustrated in Figure 1.

## Antigen Presentation in TLOs

We have recently extensively studied antigen presentation in ATLOs (72), by using the Eα-GFP/Y-Ae system to visualize antigen uptake through a GFP tag and tracking of Eα peptide/MHC-II presentation using a commercially available (Y-Ae) Ab (73–75). In the case of ATLO APCs, acquisition and presentation of soluble antigen upon MHC class II occurs within a matter of hours (72). However, unlike in the LN, presentation in the ATLO occurs equally across the major APC populations, perhaps more consistent with free diffusion of the antigen rather than transport to defined niches and compartments (72). Around 80% of the CD11c^hi^MHC-II^+^ APCs were monocyte-derived CD11b^+^DC-SIGN^+^ cells, 15% were CD11b^+^DC-SIGN*^−^* conventional DCs, and 5% CD11b*^−^*DC-SIGN*^−^* lymphoid DCs. The majority (80%) of MHC-II^+^CD11c^lo/^*^−^* APCs were CD19^+^CD11b*^−^* B cells and 10% were CD19*^−^*CD11b^+^ macrophages. 1–2% of CD11c^lo^SiglecH^+^ plasmacytoid dendritic cells (pDCs) were also detectable within the ATLO. Following Eα i.v. administration, around 55% of MHC-II^hi^Y-Ae^+^ APCs were CD11c^+^CD11b^+^DC-SIGN^+^, followed by B cells, conventional DCs, CD11c^lo/^*^−^* macrophages, and lymphoid DCs. None of the pDCs were Y-Ae^+^, in contrast with what was previously observed by us in the aorta of early atherosclerotic mice (73, 74). In summary, DCs, macrophages and B cells were the major ATLO APCs.

Altering the kinetics of antigen presentation is known to influence the outcome of T cell responses (76–81). How, or even if, differences in antigen handling between TLOs and LNs impact on the ensuing immune response is unknown. While TLOs form at sites of active antigen presentation and functional lymphocyte responses, they also support entry and priming of naïve T cells within the tissue. Intranasal delivered mature BM-derived DCs pulsed with OVApeptide (SIINFEKL) were readily detected in iBALT and able to induce proliferative responses in naïve OT-I CD8^+^ T cells (2). Similarly, OT-I T cells proliferated in tumor-associated tertiary lymphoid structures following interaction with DCs (82). Notably, multiphoton imaging revealed that the dynamics of naïve T cell migration and interaction with antigen-bearing DCs in iBALT (2) was consistent with the three phases of T cell priming reported by Mempel et al. (83). A similar observation relating to CD4^+^ T cell behavior showed OT-II T cells clustering around ATLO resident CD11c^+^ cells following antigen challenge (72), reminiscent of that seen in LN priming of CD4^+^ T cells (84). Priming of naïve T cells within ectopic structures may have beneficial or detrimental effects depending upon context of the ongoing immune response, being beneficial in infectious disease where secondary infection is a risk or contributing to epitope spreading in autoimmune disease.

Another possible role of TLOs may be the provision of a localized concentration of antigen, either from an infection or, in the case of autoimmunity, self-antigen(s). Although some transient TLOs disperse after antigen clearance, as in iBALT, this dissolution can be delayed by up to 3 weeks after the infection has resolved (2). However, it may be possible that this lag period exists due to some antigen in the form of immune complexes being displayed by FDCs. In either respect, this persistence may enable more efficient development and maintenance of memory cells, as suggested by data from ATLO and allograft studies (72, 85), and therefore a more effective *in situ* response to subsequent re-infection or antigen challenge.

## Future Directions

Clearly, many questions remain unresolved with regard to the importance and mechanism(s) of antigen presentation within TLOs, where lack of isolation or encapsulation may, in many instances, allow for a much greater degree of exposure to free antigen for all cells within the tissue. Details such as *in situ* neo-antigen availability, the timing of antigen arrival and presentation, the context in which antigen is encountered by specific cells, and the ability of those cells to receive appropriate co-stimulatory or tolerogenic signals remain to be elucidated.

With advances in cellular imaging techniques, in combination with trackable antigens and cell populations, the answers to such questions are becoming increasingly tangible. The elegant application of such approaches has successfully furthered our understanding of soluble antigen and immune complex trafficking and related immune cell interactions in SLOs (86–88). By identifying key antigen handling routes and responding cells in a dynamic setting, the possibility to develop antigen-specific therapeutics targeting TLO functions becomes a more exciting and viable option.

The identification of key antigen specificities must also be allied with such imaging approaches. The increasing power of next generation sequencing techniques makes the sequencing of both T and B cell repertoires (89, 90) in TLOs a reality. Biases in repertoire indicating clonal responses could yield valuable information pertaining to antigen specificity. At the very least, key clonal populations could be identified and used as biomarkers or even be targeted to prevent or augment antigen-specific responses as required.

## Author Contributions

All authors listed have made substantial, direct, and intellectual contribution to the work and approved it for publication.

## Conflict of Interest Statement

The authors declare that the research was conducted in the absence of any commercial or financial relationships that could be construed as a potential conflict of interest.

## References

[1] Aloisi F Pujol-Borrell R. Lymphoid neogenesis in chronic inflammatory diseases. Nat Rev Immunol (2006) 6(3):205–17. 10.1038/nri1786 16498451

[2] Halle S Dujardin HC Bakocevic N Fleige H Danzer H Willenzon S Induced bronchus-associated lymphoid tissue serves as a general priming site for T cells and is maintained by dendritic cells. J Exp Med (2009) 206(12):2593–601. 10.1084/jem.20091472 19917776 PMC2806625

[3] GeurtsvanKessel CH Willart MA Bergen IM van Rijt LS Muskens F Elewaut D Dendritic cells are crucial for maintenance of tertiary lymphoid structures in the lung of influenza virus-infected mice. J Exp Med (2009) 206(11):2339–49. 10.1084/jem.20090410 19808255 PMC2768850

[4] Jupelli M Shimada K Chiba N Slepenkin A Alsabeh R Jones HD *Chlamydia pneumoniae* infection in mice induces chronic lung inflammation, iBALT formation, and fibrosis. PLoS One (2013) 8(10):e77447. 10.1371/journal.pone.0077447 24204830 PMC3808399

[5] Grabner R Lotzer K Dopping S Hildner M Radke D Beer M Lymphotoxin beta receptor signaling promotes tertiary lymphoid organogenesis in the aorta adventitia of aged ApoE-/- mice. J Exp Med (2009) 206(1):233–48. 10.1084/jem.20080752 19139167 PMC2626665

[6] Yin C Mohanta SK Srikakulapu P Weber C Habenicht AJR Artery tertiary lymphoid organs: powerhouses of atherosclerosis immunity. Front Immunol (2016) 7:387 10.3389/fimmu.2016.00387 27777573 PMC5056324

[7] Hiraoka N Ino Y Yamazaki-Itoh R. Tertiary lymphoid organs in cancer tissues. Front Immunol (2016) 7:244. 10.3389/fimmu.2016.00244 27446075 PMC4916185

[8] Sautès-Fridman C Lawand M Giraldo NA Kaplon H Germain C Fridman WH Tertiary lymphoid structures in cancers: prognostic value, regulation, and manipulation for therapeutic intervention. Front Immunol (2016) 7:407. 10.3389/fimmu.2016.00407 27752258 PMC5046074

[9] Pitzalis C Jones GW Bombardieri M Jones SA. Ectopic lymphoid-like structures in infection, cancer and autoimmunity. Nat Rev Immunol (2014) 14(7):447–62. 10.1038/nri3700 24948366

[10] Weyand CM Goronzy JJ. Ectopic germinal center formation in rheumatoid synovitis. Ann N Y Acad Sci (2003) 987:140–9. 10.1111/j.1749-6632.2003.tb06042.x 12727633

[11] Bombardieri M Pitzalis C. Ectopic lymphoid neogenesis and lymphoid chemokines in Sjogren’s syndrome: at the interplay between chronic inflammation, autoimmunity and lymphomagenesis. Curr Pharm Biotechnol (2012) 13(10):1989–96. 10.2174/138920112802273209 22208651

[12] Serafini B Rosicarelli B Magliozzi R Stigliano E Aloisi F. Detection of ectopic B-cell follicles with germinal centers in the meninges of patients with secondary progressive multiple sclerosis. Brain Pathol (2004) 14(2):164–74. 10.1111/j.1750-3639.2004.tb00049.x 15193029 PMC8095922

[13] Tumanov A Kuprash D Lagarkova M Grivennikov S Abe K Shakhov A Distinct role of surface lymphotoxin expressed by B cells in the organization of secondary lymphoid tissues. Immunity (2002) 17(3):239–50. 10.1016/S1074-7613(02)00397-7 12354378

[14] Kratz A Campos-Neto A Hanson MS Ruddle NH. Chronic inflammation caused by lymphotoxin is lymphoid neogenesis. J Exp Med (1996) 183(4):1461–72. 10.1084/jem.183.4.1461 8666904 PMC2192477

[15] Barone F Nayar S Campos J Cloake T Withers DR Toellner KM IL-22 regulates lymphoid chemokine production and assembly of tertiary lymphoid organs. Proc Natl Acad Sci U S A (2015) 112(35):11024–9. 10.1073/pnas.1503315112 26286991 PMC4568258

[16] Marinkovic T Garin A Yokota Y Fu YX Ruddle NH Furtado GC Interaction of mature CD3+CD4+ T cells with dendritic cells triggers the development of tertiary lymphoid structures in the thyroid. J Clin Invest (2006) 116(10):2622–32. 10.1172/jci28993 16998590 PMC1570377

[17] Moyron-Quiroz JE Rangel-Moreno J Kusser K Hartson L Sprague F Goodrich S Role of inducible bronchus associated lymphoid tissue (iBALT) in respiratory immunity. Nat Med (2004) 10(9):927–34. 10.1038/nm1091 15311275

[18] Rangel-Moreno J Hartson L Navarro C Gaxiola M Selman M Randall TD. Inducible bronchus-associated lymphoid tissue (iBALT) in patients with pulmonary complications of rheumatoid arthritis. J Clin Invest (2006) 116(12):3183–94. 10.1172/jci28756 17143328 PMC1678820

[19] Jones GW Hill DG Jones SA. Understanding immune cells in tertiary lymphoid organ development: it is all starting to come together. Front Immunol (2016) 7:401. 10.3389/fimmu.2016.00401 27752256 PMC5046062

[20] Guedj K Khallou-Laschet J Clement M Morvan M Gaston AT Fornasa G M1 macrophages act as LTβR-independent lymphoid tissue inducer cells during atherosclerosis-related lymphoid neogenesis. Cardiovasc Res (2014) 101(3):434–43. 10.1093/cvr/cvt263 24272771

[21] McDonald KG McDonough JS Newberry RD. Adaptive immune responses are dispensable for isolated lymphoid follicle formation: antigen-naive, lymphotoxin-sufficient B lymphocytes drive the formation of mature isolated lymphoid follicles. J Immunol (2005) 174(9):5720–8. 10.4049/jimmunol.174.9.5720 15843574

[22] Ruddle NH. Lymphatic vessels and tertiary lymphoid organs. J Clin Invest (2014) 124(3):953–9. 10.1172/jci71611 24590281 PMC3934190

[23] Kambayashi T Laufer TM. Atypical MHC class II-expressing antigen-presenting cells: can anything replace a dendritic cell? Nat Rev Immunol (2014) 14(11):719–30. 10.1038/nri3754 25324123

[24] Muniz LR Pacer ME Lira SA Furtado GC. A critical role for dendritic cells in the formation of lymphatic vessels within tertiary lymphoid structures. J Immunol (2011) 187(2):828–34. 10.4049/jimmunol.1004233 21666055 PMC3137511

[25] Browning JL Allaire N Ngam-Ek A Notidis E Hunt J Perrin S Lymphotoxin-beta receptor signaling is required for the homeostatic control of HEV differentiation and function. Immunity (2005) 23(5):539–50. 10.1016/j.immuni.2005.10.002 16286021

[26] Ludewig B Odermatt B Landmann S Hengartner H Zinkernagel RM. Dendritic cells induce autoimmune diabetes and maintain disease *via* de novo formation of local lymphoid tissue. J Exp Med (1998) 188(8):1493–501. 10.1084/jem.188.8.1493 9782126 PMC2213416

[27] Dieu-Nosjean MC Antoine M Danel C Heudes D Wislez M Poulot V Long-term survival for patients with non-small-cell lung cancer with intratumoral lymphoid structures. J Clin Oncol (2008) 26(27):4410–7. 10.1200/JCO.2007.15.0284 18802153

[28] Germain C Gnjatic S Tamzalit F Knockaert S Remark R Goc J Presence of B cells in tertiary lymphoid structures is associated with a protective immunity in patients with lung cancer. Am J Respir Crit Care Med (2014) 189(7):832–44. 10.1164/rccm.201309-1611OC 24484236

[29] Cho HJ Shashkin P Gleissner CA Dunson D Jain N Lee JK Induction of dendritic cell-like phenotype in macrophages during foam cell formation. Physiol Genomics (2007) 29(2):149–60. 10.1152/physiolgenomics.00051.2006 17244792

[30] Chen X Jensen PE. The role of B lymphocytes as antigen-presenting cells. Arch Immunol Ther Exp (2008) 56(2):77–83. 10.1007/s00005-008-0014-5 18373241

[31] Srikakulapu P Hu D Yin C Mohanta SK Bontha SV Peng L Artery tertiary lymphoid organs control multilayered territorialized atherosclerosis B-cell responses in aged ApoE-/- mice. Arterioscler Thromb Vasc Biol (2016) 36(6):1174–85. 10.1161/ATVBAHA.115.306983 27102965 PMC4894775

[32] Germain C Gnjatic S Dieu-Nosjean MC. Tertiary lymphoid structure-associated B cells are key players in anti-tumor immunity. Front Immunol (2015) 6:67. 10.3389/fimmu.2015.00067 25755654 PMC4337382

[33] Jones GW Jones SA. Ectopic lymphoid follicles: inducible centres for generating antigen-specific immune responses within tissues. Immunology (2016) 147(2):141–51. 10.1111/imm.12554 26551738 PMC4717241

[34] MacLennan IC. Germinal centers. Annu Rev Immunol (1994) 12:117–39. 10.1146/annurev.iy.12.040194.001001 8011279

[35] Fu YX Huang G Wang Y Chaplin DD. B lymphocytes induce the formation of follicular dendritic cell clusters in a lymphotoxin alpha-dependent fashion. J Exp Med (1998) 187(7):1009–18. 10.1084/jem.187.7.1009 9529317 PMC2212211

[36] Allen CD Cyster JG. Follicular dendritic cell networks of primary follicles and germinal centers: phenotype and function. Semin Immunol (2008) 20(1):14–25. 10.1016/j.smim.2007.12.001 18261920 PMC2366796

[37] Ansel KM Ngo VN Hyman PL Luther SA Forster R Sedgwick JD A chemokine-driven positive feedback loop organizes lymphoid follicles. Nature (2000) 406(6793):309–14. 10.1038/35018581 10917533

[38] Le Pottier L Devauchelle V Fautrel A Daridon C Saraux A Youinou P Ectopic germinal centers are rare in Sjogren’s syndrome salivary glands and do not exclude autoreactive B cells. J Immunol (2009) 182(6):3540–7. 10.4049/jimmunol.0803588 19265132

[39] Heesters BA Myers RC Carroll MC. Follicular dendritic cells: dynamic antigen libraries. Nat Rev Immunol (2014) 14(7):495–504. 10.1038/nri3689 24948364

[40] Kranich J Krautler NJ. How follicular dendritic cells shape the B-cell antigenome. Front Immunol (2016) 7:225. 10.3389/fimmu.2016.00225 27446069 PMC4914831

[41] Victoratos P Kollias G. Induction of autoantibody-mediated spontaneous arthritis critically depends on follicular dendritic cells. Immunity (2009) 30(1):130–42. 10.1016/j.immuni.2008.10.019 19119026

[42] Drayton DL Ying X Lee J Lesslauer W Ruddle NH. Ectopic LT alpha beta directs lymphoid organ neogenesis with concomitant expression of peripheral node addressin and a HEV-restricted sulfotransferase. J Exp Med (2003) 197(9):1153–63. 10.1084/jem.20021761 12732657 PMC2193975

[43] Moser B Schaerli P Loetscher P CXCR5(+) T cells: follicular homing takes center stage in T-helper-cell responses. Trends Immunol (2002) 23(5):250–4. 10.1016/S1471-4906(02)02218-4 12102746

[44] Barone F Bombardieri M Manzo A Blades MC Morgan PR Challacombe SJ Association of CXCL13 and CCL21 expression with the progressive organization of lymphoid-like structures in Sjogren’s syndrome. Arthritis Rheum (2005) 52(6):1773–84. 10.1002/art.21062 15934082

[45] Stranford S Ruddle NH. Follicular dendritic cells, conduits, lymphatic vessels, and high endothelial venules in tertiary lymphoid organs: parallels with lymph node stroma. Front Immunol (2012) 3:350. 10.3389/fimmu.2012.00350 23230435 PMC3515885

[46] Fleige H Ravens S Moschovakis GL Bölter J Willenzon S Sutter G IL-17-induced CXCL12 recruits B cells and induces follicle formation in BALT in the absence of differentiated FDCs. J Exp Med (2014) 211(4):643–51. 10.1084/jem.20131737 24663215 PMC3978277

[47] El Shikh ME Pitzalis C. Follicular dendritic cells in health and disease. Front Immunol (2012) 3:292. 10.3389/fimmu.2012.00292 23049531 PMC3448061

[48] Ishimaru N Arakaki R Yoshida S Yamada A Noji S Hayashi Y Expression of the retinoblastoma protein RbAp48 in exocrine glands leads to Sjogren’s syndrome-like autoimmune exocrinopathy. J Exp Med (2008) 205(12):2915–27. 10.1084/jem.20080174 19015307 PMC2585852

[49] Kapsogeorgou EK Moutsopoulos HM Manoussakis MN Functional expression of a costimulatory B7.2 (CD86) protein on human salivary gland epithelial cells that interacts with the CD28 receptor, but has reduced binding to CTLA4. J Immunol (2001) 166(5):3107–13. 10.4049/jimmunol.166.5.3107 11207262

[50] Dimitriou ID Kapsogeorgou EK Moutsopoulos HM Manoussakis MN CD40 on salivary gland epithelial cells: high constitutive expression by cultured cells from Sjogren’s syndrome patients indicating their intrinsic activation. Clin Exp Immunol (2002) 127(2):386–92. 10.1046/j.1365-2249.2002.01752.x 11876766 PMC1906327

[51] Tsunawaki S Nakamura S Ohyama Y Sasaki M Ikebe-Hiroki A Hiraki A Possible function of salivary gland epithelial cells as nonprofessional antigen-presenting cells in the development of Sjogren’s syndrome. J Rheumatol (2002) 29(9):1884–96. 12233883

[52] Londei M Lamb JR Bottazzo GF Feldmann M. Epithelial cells expressing aberrant MHC class II determinants can present antigen to cloned human T cells. Nature (1984) 312(5995):639–41. 10.1038/312639a0 6334239

[53] Cohen JN Guidi CJ Tewalt EF Qiao H Rouhani SJ Ruddell A Lymph node-resident lymphatic endothelial cells mediate peripheral tolerance *via* Aire-independent direct antigen presentation. J Exp Med (2010) 207(4):681–8. 10.1084/jem.20092465 20308365 PMC2856027

[54] Gardner JM Devoss JJ Friedman RS Wong DJ Tan YX Zhou X Deletional tolerance mediated by extrathymic Aire-expressing cells. Science (2008) 321(5890):843–7. 10.1126/science.1159407 18687966 PMC2532844

[55] van Delft MA Huitema LF Tas SW. The contribution of NF-κB signalling to immune regulation and tolerance. Eur J Clin Invest (2015) 45(5):529–39. 10.1111/eci.12430 25735405

[56] Fletcher AL Lukacs-Kornek V Reynoso ED Pinner SE Bellemare-Pelletier A Curry MS Lymph node fibroblastic reticular cells directly present peripheral tissue antigen under steady-state and inflammatory conditions. J Exp Med (2010) 207(4):689–97. 10.1084/jem.20092642 20308362 PMC2856033

[57] Link A Hardie DL Favre S Britschgi MR Adams DH Sixt M Association of T-zone reticular networks and conduits with ectopic lymphoid tissues in mice and humans. Am J Pathol (2011) 178(4):1662–75. 10.1016/j.ajpath.2010.12.039 21435450 PMC3070229

[58] Bajenoff M Granjeaud S Guerder S. The strategy of T cell antigen-presenting cell encounter in antigen-draining lymph nodes revealed by imaging of initial T cell activation. J Exp Med (2003) 198(5):715–24. 10.1084/jem.20030167 12953093 PMC2194192

[59] Sixt M Kanazawa N Selg M Samson T Roos G Reinhardt DP The conduit system transports soluble antigens from the afferent lymph to resident dendritic cells in the T cell area of the lymph node. Immunity (2005) 22(1):19–29. 10.1016/j.immuni.2004.11.013 15664156

[60] Roozendaal R Mempel TR Pitcher LA Gonzalez SF Verschoor A Mebius RE Conduits mediate transport of low-molecular-weight antigen to lymph node follicles. Immunity (2009) 30(2):264–76. 10.1016/j.immuni.2008.12.014 19185517 PMC2699624

[61] Bajenoff M Germain RN. B-cell follicle development remodels the conduit system and allows soluble antigen delivery to follicular dendritic cells. Blood (2009) 114(24):4989–97. 10.1182/blood-2009-06-229567 19713459 PMC2788973

[62] Clark SL Jr The reticulum of lymph nodes in mice studied with the electron microscope. Am J Anat (1962) 110:217–57. 10.1002/aja.1001100303 13879732

[63] Pape KA Catron DM Itano AA Jenkins MK. The humoral immune response is initiated in lymph nodes by B cells that acquire soluble antigen directly in the follicles. Immunity (2007) 26(4):491–502. 10.1016/j.immuni.2007.02.011 17379546

[64] Carrasco YR Batista FD. B cells acquire particulate antigen in a macrophage-rich area at the boundary between the follicle and the subcapsular sinus of the lymph node. Immunity (2007) 27(1):160–71. 10.1016/j.immuni.2007.06.007 17658276

[65] Junt T Moseman EA Iannacone M Massberg S Lang PA Boes M Subcapsular sinus macrophages in lymph nodes clear lymph-borne viruses and present them to antiviral B cells. Nature (2007) 450(7166):110–4. 10.1038/nature06287 17934446

[66] Phan TG Grigorova I Okada T Cyster JG. Subcapsular encounter and complement-dependent transport of immune complexes by lymph node B cells. Nat Immunol (2007) 8(9):992–1000. 10.1038/ni1494 17660822

[67] Qi H Egen JG Huang AY Germain RN. Extrafollicular activation of lymph node B cells by antigen-bearing dendritic cells. Science (2006) 312(5780):1672–6. 10.1126/science.1125703 16778060

[68] Delamarre L Pack M Chang H Mellman I Trombetta ES. Differential lysosomal proteolysis in antigen-presenting cells determines antigen fate. Science (2005) 307(5715):1630–4. 10.1126/science.1108003 15761154

[69] Penaranda C Tang Q Ruddle NH Bluestone JA. Prevention of diabetes by FTY720-mediated stabilization of peri-islet tertiary lymphoid organs. Diabetes (2010) 59(6):1461–8. 10.2337/db09-1129 20299465 PMC2874707

[70] Kraal G. Cells in the marginal zone of the spleen. Int Rev Cytol (1992) 132:31–74. 10.1016/S0074-7696(08)62453-5 1555921

[71] Nolte MA Belien JA Schadee-Eestermans I Jansen W Unger WW van Rooijen N A conduit system distributes chemokines and small blood-borne molecules through the splenic white pulp. J Exp Med (2003) 198(3):505–12. 10.1084/jem.20021801 12900524 PMC2194088

[72] Hu D Mohanta SK Yin C Peng L Ma Z Srikakulapu P Artery tertiary lymphoid organs control aorta immunity and protect against atherosclerosis *via* vascular smooth muscle cell lymphotoxin beta receptors. Immunity (2015) 42(6):1100–15. 10.1016/j.immuni.2015.05.015 26084025 PMC4678289

[73] Macritchie N Grassia G Sabir SR Maddaluno M Welsh P Sattar N Plasmacytoid dendritic cells play a key role in promoting atherosclerosis in apolipoprotein E-deficient mice. Arterioscler Thromb Vasc Biol (2012) 32(11):2569–79. 10.1161/ATVBAHA.112.251314 22936340

[74] Sage AP Murphy D Maffia P Masters LM Sabir SR Baker LL MHC Class II-restricted antigen presentation by plasmacytoid dendritic cells drives proatherogenic T cell immunity. Circulation (2014) 130(16):1363–73. 10.1161/CIRCULATIONAHA.114.011090 25223984 PMC4428652

[75] Maddaluno M MacRitchie N Grassia G Ialenti A Butcher JP Garside P Murine aortic smooth muscle cells acquire, though fail to present exogenous protein antigens on major histocompatibility complex class II molecules. Biomed Res Int (2014) 2014:949845. 10.1155/2014/949845 25136640 PMC4127268

[76] Obst R van Santen HM Melamed R Kamphorst AO Benoist C Mathis D. Sustained antigen presentation can promote an immunogenic T cell response, like dendritic cell activation. Proc Natl Acad Sci U S A (2007) 104(39):15460–5. 10.1073/pnas.0707331104 17881563 PMC2000557

[77] Celli S Lemaitre F Bousso P. Real-time manipulation of T cell-dendritic cell interactions in vivo reveals the importance of prolonged contacts for CD4+ T cell activation. Immunity (2007) 27(4):625–34. 10.1016/j.immuni.2007.08.018 17950004

[78] Baumjohann D Preite S Reboldi A Ronchi F Ansel KM Lanzavecchia A Persistent antigen and germinal center B cells sustain T follicular helper cell responses and phenotype. Immunity (2013) 38(3):596–605. 10.1016/j.immuni.2012.11.020 23499493

[79] Henrickson SE Perro M Loughhead SM Senman B Stutte S Quigley M Antigen availability determines CD8(+) T cell-dendritic cell interaction kinetics and memory fate decisions. Immunity (2013) 39(3):496–507. 10.1016/j.immuni.2013.08.034 24054328 PMC3914670

[80] Rabenstein H Behrendt AC Ellwart JW Naumann R Horsch M Beckers J Differential kinetics of antigen dependency of CD4+ and CD8+ T cells. J Immunol (2014) 192(8):3507–17. 10.4049/jimmunol.1302725 24639353

[81] Benson RA MacLeod MK Hale BG Patakas A Garside P Brewer JM. Antigen presentation kinetics control T cell/dendritic cell interactions and follicular helper T cell generation in vivo. Elife (2015) 4. 10.7554/eLife.06994 26258879 PMC4558563

[82] Joshi NS Akama-Garren EH Lu Y Lee DY Chang GP Li A Regulatory T cells in tumor-associated tertiary lymphoid structures suppress anti-tumor T cell responses. Immunity (2015) 43(3):579–90. 10.1016/j.immuni.2015.08.006 26341400 PMC4826619

[83] Mempel TR Henrickson SE Von Andrian UH. T-cell priming by dendritic cells in lymph nodes occurs in three distinct phases. Nature (2004) 427(6970):154–9. 10.1038/nature02238 14712275

[84] Zinselmeyer BH Dempster J Gurney AM Wokosin D Miller M Ho H In situ characterization of CD4+ T cell behavior in mucosal and systemic lymphoid tissues during the induction of oral priming and tolerance. J Exp Med (2005) 201(11):1815–23. 10.1084/jem.20050203 15928201 PMC2213276

[85] Nasr IW Reel M Oberbarnscheidt MH Mounzer RH Baddoura FK Ruddle NH Tertiary lymphoid tissues generate effector and memory T cells that lead to allograft rejection. Am J Transplant (2007) 7(5):1071–9. 10.1111/j.1600-6143.2007.01756.x 17359505

[86] Itano AA Jenkins MK. Antigen presentation to naive CD4 T cells in the lymph node. Nat Immunol (2003) 4(8):733–9. 10.1038/ni957 12888794

[87] Cyster JG. B cell follicles and antigen encounters of the third kind. Nat Immunol (2010) 11(11):989–96. 10.1038/ni.1946 20959804

[88] Germain RN Robey EA Cahalan MD. A decade of imaging cellular motility and interaction dynamics in the immune system. Science (2012) 336(6089):1676–81. 10.1126/science.1221063 22745423 PMC3405774

[89] Robins H. Immunosequencing: applications of immune repertoire deep sequencing. Curr Opin Immunol (2013) 25(5):646–52. 10.1016/j.coi.2013.09.017 24140071

[90] Calis JJ Rosenberg BR. Characterizing immune repertoires by high throughput sequencing: strategies and applications. Trends Immunol (2014) 35(12):581–90. 10.1016/j.it.2014.09.004 25306219 PMC4390416

